# Aerobic fitness mediates the intervention effects of a school-based physical activity intervention on academic performance. The school in Motion study – A cluster randomized controlled trial

**DOI:** 10.1016/j.pmedr.2021.101648

**Published:** 2021-11-24

**Authors:** Runar Barstad Solberg, Jostein Steene-Johannessen, Morten Wang Fagerland, Sigmund A. Anderssen, Sveinung Berntsen, Geir K. Resaland, Esther M.F. van Sluijs, Ulf Ekelund, Elin Kolle

**Affiliations:** a Department of Sports Medicine, Norwegian School of Sport Sciences, PB 4014, Ullevål Stadion, 0806 Oslo, Norway; b UKCRC Centre for Diet and Activity Research (CEDAR) and MRC Epidemiology Unit, School of Clinical Medicine, University of Cambridge, Cambridge CB2 0QQ, UK; c Faculty of Health and Sport Science, Department of Sport Science and Physical Education, University of Agder, PB 422, 4604 Kristiansand, Norway; d Center for Physically Active Learning, Faculty of Education, Arts and Sports, Western Norway University of Applied Sciences, Campus Sogndal, 6856 Sogndal, Norway

**Keywords:** ScIM, School in Motion, PAL, Physically Active Learning, DWBH, Don’t worry – be Happy, SD, standard deviation, ICC, intra class correlation, Physical activity, Aerobic fitness, Academic performance, Cluster RCT, Adolescents

## Abstract

Little information exists on the mechanism of how physical activity interventions effects academic performance. We examined whether the effects of a school-based physical activity intervention on academic performance were mediated by aerobic fitness. The School in Motion study was a nine-month cluster randomized controlled trial between September 2017 and June 2018. Students from 30 Norwegian lower secondary schools (*N* = 2,084, mean age [*SD*] = 14 [0.3] years) were randomly assigned into three groups: the Physically Active Learning (PAL) intervention (*n* = 10), the Don’t Worry—Be Happy (DWBH) intervention (*n* = 10), or control (*n* = 10). Aerobic fitness was assessed by the Andersen test and academic performance by national tests in reading and numeracy. Mediation was assessed according to the causal steps approach using linear mixed models. In the PAL intervention, aerobic fitness partially mediated the intervention effect on numeracy by 28% from a total effect of 1.73 points (95% CI: 1.13 to 2.33) to a natural direct effect of 1.24 points (95% CI: 0.58 to 1.91), and fully mediated the intervention effect on reading, with the total effect of 0.89 points (95% CI: 0.15 to 1.62) reduced to the natural direct effect of 0.40 points (95% CI: −0.48 to 1.28). Aerobic fitness did not mediate the effects on academic performance in the DWBH intervention. As aerobic fitness mediated the intervention effect on academic performance in one intervention, physical activity of an intensity that increases aerobic fitness is one strategy to improve academic performance among adolescents.

## Highlights

• Physical activity is associated with increased aerobic fitness and academic performance.
• Little is known on mechanism of physical activity effects on academic performance.
• We performed a cluster randomized controlled trial.
• Aerobic fitness mediated the intervention effect on academic performance.
• Activity increasing aerobic fitness is a strategy to improve academic performance.

## 1 Introduction

Physical activity (PA) and aerobic fitness is associated with several health benefits in youths (Poitras et al., 2016, Raghuveer et al., 2020). Worryingly, accelerometer data shows that PA levels decline throughout adolescents (van Sluijs et al., 2021) and a recent systematic review reported a decline in children and adolescents aerobic fitness over the past three decades (Tomkinson et al., 2019). Interventions aimed at increasing PA levels and aerobic fitness among adolescents are therefore warranted.

Schools is an ideal avenue for health promoting interventions cause you reach individuals from all backgrounds. Hence, numerous school-based PA interventions aimed at increasing children and adolescents PA and aerobic fitness have been developed (Hartwig et al., 2021, Love et al., 2019). Emerging evidence shows positive associations between PA, aerobic fitness and academic performance (Marques et al., 2018, Santana et al., 2017), making PA interventions relevant for schools, teachers and stakeholders. However, whilst the most recent systematic review on the effects of school-based PA intervention on academic performance reports strong evidence for the favourable effects on numeracy performance, the evidence of effects on overall academic performance is inconclusive (Singh et al., 2019). The fact that many studies do not show any effect of PA interventions on academic performance may be explained by failure of most school-based interventions to increase children and adolescents’ PA level (Love et al., 2019). The inconsistent findings in the literature call attention to the lack of knowledge regarding through which mechanisms school-based PA intervention may enhance academic performance.

Several potential mechanisms have been suggested in the literature. One mechanism suggest that higher PA levels leads to increased neurogenesis in hippocampus associated with learning and memory (van Praag, 2008), increases in important growth factors leading to a variety of structural brain changes (Lubans et al., 2016), and higher levels of executive functions such as inhibition and working memory (Hillman et al., 2011). Another mechanism suggests that positive effects of increased PA on academic performance is mediated through aerobic fitness (Raghuveer et al., 2020). Increased PA of a certain intensity leads to improved aerobic fitness (Raghuveer et al., 2020) which can affect brain morphology (Chaddock et al., 2011) and thus improve executive functions and academic performance (Marques et al., 2018).

In a recent cross sectional study, aerobic fitness mediated the associations between PA and academic performance among 186 Spanish children 9–11 year-olds (Visier-Alfonso et al., 2021). This corresponds with findings among 608 Japanese seventh graders where it was suggested that aerobic fitness meditated the associations between PA and academic performance among boys but not girls (Kyan et al., 2019). Also, in a study of 401 American children in second and third grade, aerobic fitness mediated the association between PA and numeracy but not reading (Lambourne et al., 2013). However, a study of 232 Swedish adolescents did not support these findings (Kwak et al., 2009). The cross-sectional design used in the mentioned studies precludes casual interpretation, and there is need for intervention studies examining the mediating role of aerobic fitness on effects of school-based PA interventions on academic performance.

We conducted a cluster randomized controlled trial (RCT) with two different PA interventions including more than 2,000 Norwegian 14-year-olds in 30 lower secondary schools titled the School in Motion (ScIM) study. The results revealed that students in both intervention arms significantly improved academic performance in numeracy and reading compared to students in the control group (Solberg et al., 2021), and students in one of the intervention arm also significantly improved accelerometer assessed PA levels (primary outcome) and aerobic fitness compared with controls (Kolle et al., 2020). The study design allows us to investigate whether aerobic fitness is on the causal pathway between increased PA and the intervention effects on academic performance. By performing mediation analysis, we can evaluate the possibility that an exposure variable causes changes in the mediator variable, which in turn causes the outcome variable to change (Valeri and Vanderweele, 2013). Further, the design in ScIM will reduce potential confounding observed with cross-sectional studies and contribute knowledge on which mechanism is important to enhance academic performance.

The aim of the present study was to examine the mediating role of aerobic fitness on the intervention effect of a school-based PA intervention on academic performance in 14-year-olds.

## 2 Methods

The ScIM study was a nine-month cluster RCT of 2,084 14-year-olds from 30 lower secondary schools in Norway. Schools were randomly allocated in a 1:1:1 ratio to either the Physically Active Learning (PAL) intervention (*n* = 10), the Don’t Worry-Be Happy (DWBH) intervention (*n* = 10), or control (*n* = 10). One school withdrew after randomization but prior to baseline testing, leaving nine schools in the control group. The project was reviewed by the Regional Committee for Medical and Health Research Ethics (REK) in Norway, who according to the act on medical and health research (the Health Research Act 2008), concluded that the study did not require full review by REK. The ScIM study was approved by the Norwegian Centre for Research Data and adhered to the Helsinki Declaration (2008). Parents gave their written informed consent allowing their adolescents to participate. This content could be revoked by the parents or adolescents at any time. The ScIM is registered in ClinicalTrials.gov (25/01/2019), ID nr: NCT03817047. The design, conduct, and reporting of this trial follow recommendations of the CONSORT statement. The CONSORT checklist can be found in Supplementary File 1. The methodology and main effects of the ScIM study have been described in detail elsewhere (Kolle et al., 2020). A brief description is provided below.

### 2.1 The ScIM interventions

Both interventions were based on a socio-ecological framework that recognizes the complex interplay between personal and environmental influences on behavior (McLeroy et al., 1988) and provided approximately 120 min of additional PA in addition to the mandatory 120–180 min of physical education (PE), lessons per week. The interventions were delivered from September 2017 to June 2018, and intervention components were mandatory for all students. Control schools continued the current practice with the usual amount of mandatory PE and were asked not to implement additional PA in the curriculum.

The PAL intervention focused on increasing the students’ PA levels and consisted of three components of at least moderate intensity: (1) additional lesson of PE per week (60 min); (2) 30 min/week lesson of physically active learning where physical activities were integrated in regular subjects; and (3) 30 min/week lesson of PA that included a variety of enjoyable activities. In contrast, the DWBH intervention's focus was to promote friendship through PA and consisted of two components: (1) 60 min of PE called the ‘Don’t worry’ lesson (DW) and (2) a 60 min ‘Be happy’ lesson (BH). Based on PA interest, students formed groups of 3–8 students and chose one activity that was performed throughout the intervention period.

In the PAL intervention the components were led by teachers, while the DWBH intervention was led and organized by the students themselves.

### 2.2 Measurements

Measurements were taken at baseline (April–August 2017) and in the last phase of the intervention (April–June 2018). The test procedures were identical at both time points. Data were collected at the respective schools, and all test personnel were trained by members of the research team.

#### 2.2.1 Academic performance

Numeracy and reading performance were measured using standardized computer-based national tests designed and administered by the Norwegian Directorate for Education and Training. Both tests included anchor questions, which made it possible to provide a baseline for an equating analysis between the two time points. The scores were standardized to a mean of 50 scale points with a standard deviation of 10 at each time point.

#### 2.2.2 Aerobic fitness

We used the Andersen test to assess the students’ aerobic fitness (Andersen et al., 2008). The Andersen test is an intermittent 10-minute running field test which is a reliable and valid test for determination of aerobic fitness on a group level among 10-year old children (Aadland et al., 2014). We administered the Andersen test as per standard procedures indoors on a wooden or rubber floor, however, due to different sizes of available facilities, we standardized the length to 16 m (original protocol 20 m). The test required the students to run back and forth between the two lines, with 15-second work periods and 15-second breaks standing still. Each time the students turned around at the end line, they had to touch the floor with one hand. Students were meant to run to voluntary exhaustion. Test personnel subjectively judged whether the student completed a valid test (whether the students worked hard enough) and recorded the distance covered. The distance covered (in metres) was used as a proxy for aerobic fitness.

#### 2.2.3 Anthropometry

We measured weight to the nearest 0.1 kg using a Seca 899 scale and height to the nearest 0.1 cm using a Seca 123 Portable Stadiometer (Seca, Hamburg, Germany). To account for clothing, we subtracted 0.6 kg (light clothing; gym shorts and t-shirt) or 1.5 kg (normal clothes; trousers and jumper) from the body weight measurements. Body mass index (BMI) was calculated as weight (kg) divided by the height squared (m^2^).

### 2.3 Statistical analysis

Continues outcome variables were assessed for normality and homogeneity of variance. Descriptive data are presented as mean and standard deviation (SD) unless otherwise stated. Baseline differences between participants in the three study arms were investigated using linear regressions adjusted for gender.

Prior to testing for mediation, we fitted linear mixed models to evaluate between-group differences in change from baseline to follow-up between participants in the interventions compared with controls (i.e intervention effect) for numeracy and reading performance (primary outcomes) and aerobic fitness (mediator) separately. Each model was adjusted for gender and contained fixed effects for intervention, time (from baseline to follow-up), and intervention × time interaction. We added random effects for school, in addition to class and subject ID, to accommodate for clustering within these units. Missing values were handled by the linear mixed models, so that participants with missing values in any of the variables were included in the analyses, as long as they had at least one measurement of the outcome variable. Intraclass Correlation Coefficients (ICC) for the school cluster are predicted using Statas *iccvar* command following each linear mixed model (Hedges et al., 2012).

**Fig. 1:**
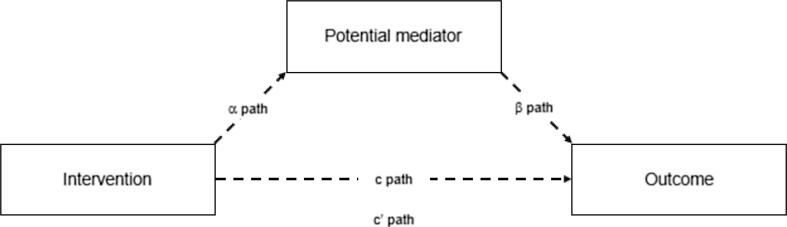
The hypothesized mediation model. c path: Intervention effect (mean difference in change between intervention and control) on outcome (the total effect). α path: Intervention effect (mean difference in change between intervention and control) on mediator of interest. β path: Association between mediator and outcome adjusted for group allocation c’ path: The natural direct intervention effect on outcome conditional on holding the mediator variable constant.

Mediation analysis was performed using four stage linear regression models with the approach in Fig. 1 (Lee et al., 2019, Valeri and Vanderweele, 2013, VanderWeele, 2016). First, the between-group differences in change from baseline to follow-up between participants in the interventions compared with controls (i.e intervention effect) on primary outcomes were assessed individually to generate the ‘total effect’ (c path). Second, the intervention effect on the hypothesized mediator was assessed (α path). Third, the association between mediators and primary outcomes adjusted for group allocation (interventions) was assessed individually (β path). Finally, we generated the natural direct effect by estimating the intervention effect on primary outcomes conditional on holding the mediator variable constant (c‘ path) and consequently generating the natural direct effect.

The natural direct effect (c‘ path) refers to the relationship between two variables that is mediated by a third variable on the pathway. In order to meet the criteria for mediation, paths α and β have to be significant with a confidence interval not crossing zero (Valeri and Vanderweele, 2013). If the exposure coefficient of the total effect (c path) is considerably different compared with the natural direct effect (c’ path), the difference could be interpreted as mediation (VanderWeele, 2016). Partial mediation is present if the natural direct effect is significant, and full mediation is present when it is attenuated and no longer significant. The total and natural direct effects were estimated with 95% confidence intervals (CI) obtained by means of the bootstrap re-sampling method with 1000 replications. Results are expressed as unstandardized, baseline-adjusted coefficients for primary outcomes (points) and mediator (meters covered) with corresponding 95% CI.

Due to differential intervention effect on academic performance for boys and girls (p < 0.001 for interaction), analysis was repeated stratified by gender. All statistical analyses were performed in Stata 16.0/SE (StataCorp LP), and the level of significance was set at *p* < 0.05.

## 3 Results

**Fig. 2:**
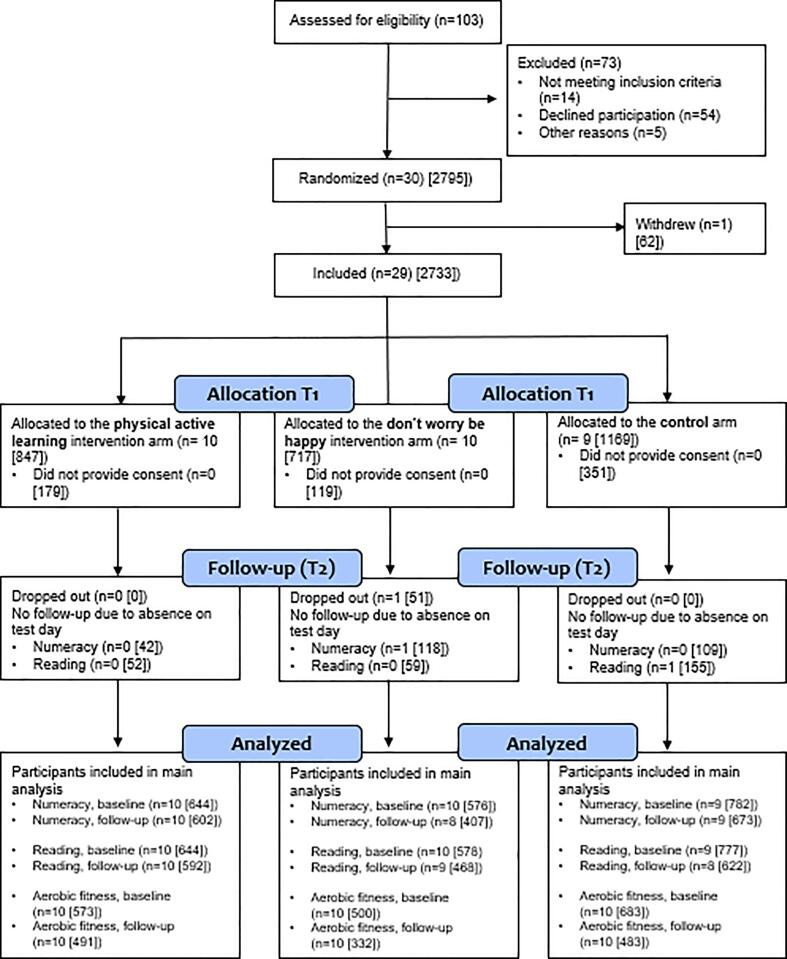
Flow diagram of the included students (n = schools [students]).

Of the 2,733 students invited to participate, 2,084 (76%) agreed to partake in the study. Of these, 1,999 students provided valid data on both outcomes (reading and numeracy) at baseline, and 1,682 students had valid data at follow-up. A total of 1,756 and 1,306 students provided valid assessment of aerobic fitness at baseline and follow-up, respectively, and were included in the analyses (Fig. 2).

At baseline, students in the PAL and DWBH had lower aerobic fitness (34 m and 21 m respectively) compared with their control school counterparts (p < 0.002), no differences were found for academic performance when comparing students in the intervention groups with students in the control group (p > 0.05 for all) (Table 1).

**Table 1:** Participants demographic and anthropometric characteristics by group allocation at baseline and follow-up.

		PAL Intervention (n = 655 – 491)				DWBH Intervention (n = 586 – 332)				Control (n = 795 – 483)	
		Baseline	Follow-up			Baseline	Follow-up			Baseline	Follow-up
		Mean (SD)	Mean (SD)			Mean (SD)	Mean (SD)			Mean (SD)	Mean (SD)
Girls/Boys (%)		50/50	50/50			50/50	50/50			50/50	50/50
Age (year)		13.9 (0.3)	14.9 (0.3)			14.0 (0.3)	14.9 (0.3)			14.0 (0.3)	14.9 (0.3)
**Anthropometry**											
Height (cm)		164.6 (8.1)	168 (8.3)			166.4 (7.7)	170 (7.9)			165.8 (7.7)	169.7 (7.8)
Weight (kg)		54.2 (10.8)	58.2 (10.9)			56.2 (11.0)	59.9 (10.7)			54.4 (10.5)	58.2 (11.2)
BMI		19.9 (3.1)	20.5 (3.2)			20.2 (3.2)	20.8 (3.0)			19.7 (3.1)	20.1 (3.1)
**Aerobic fitness**											
Aerobic fitness (m)		894 (101)	925 (108)			909 (111)	909 (90)			928 (102)	940 (92)
**Academic performance**											
Numeracy (points)		54.9 (10)	55.2 (9)			54.5 (9)	55.0 (9)			55.2 (9)	53.8 (9)
Reading (points)		55.2 (9)	54.7 (9)			54.2 (9)	54.0 (8)			54.8 (10)	53.5 (9)

PAL = Physical active learning; DWBH = Don’t worry – Be happy”, M = meter. BMI = body mass index.

**Fig. 3:**
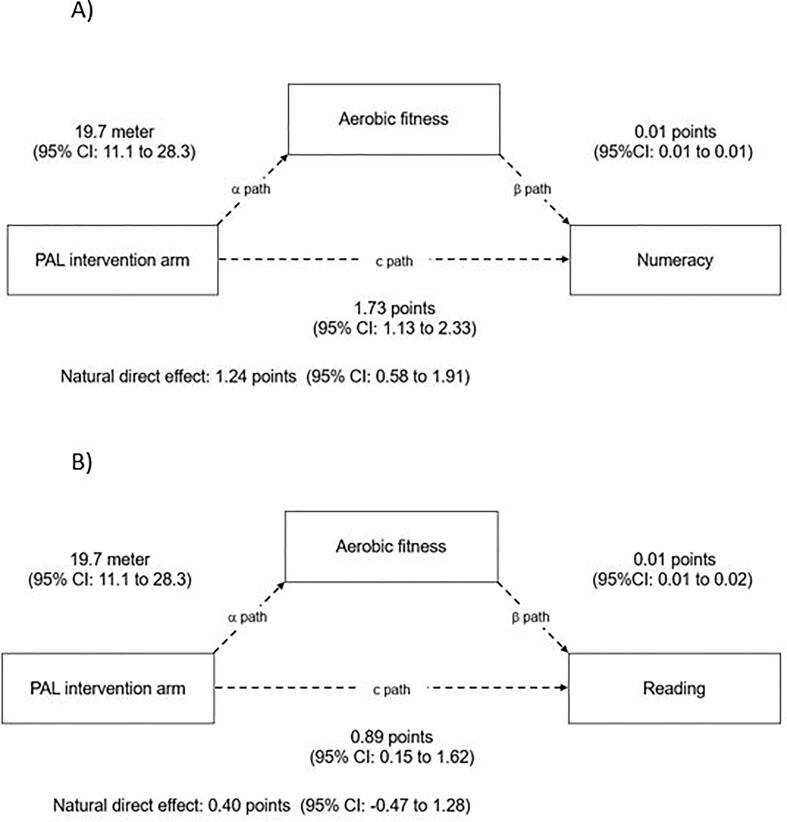
Models of the mediation effect of aerobic fitness on the intervention effect (mean difference in change (c and c’ path)) onA) numeracy and B) reading performance among students in the Physically Active Learning (PAL) intervention arm when compared with controls. All coefficients are unstandardized. Each model contained fixed effects for intervention, time (baseline – follow-up) and intervention × time interaction, in addition to random effects for school, class and subject ID. All models are adjusted for gender. CI: Confidence interval. Intra Class Correlation Coefficient for school (ICC)s: Model A: ICC: 0.04, Model B: ICC: 0.09.

**Fig. 4:**
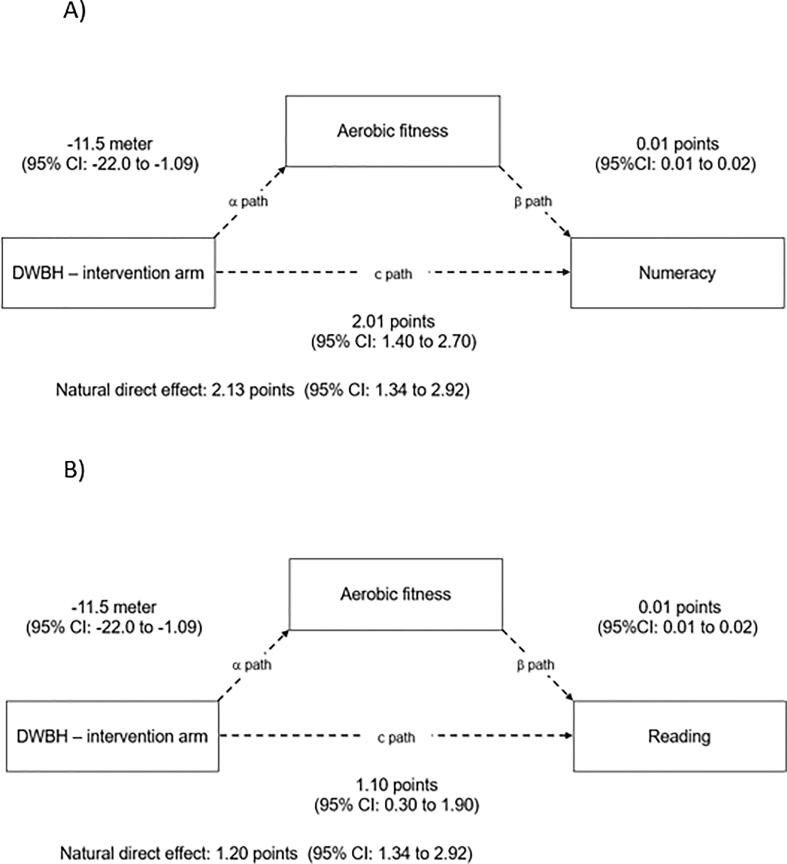
Models of the mediation effect of aerobic fitness on the intervention effect (mean difference in change (c and c’ path)) on A) numeracy and B) reading performance among students in the Don’t worry – be happy (DWBH) intervention arm when compared with controls. All coefficients are unstandardized. Each model contained fixed effects for intervention, time (baseline – follow-up) and intervention × time interaction, in addition to random effects for school, class and subject ID. All models are adjusted for gender. CI: Confidence interval. Intra Class Correlation Coefficient for school (ICC)s: Model A: ICC: 0.08, Model B: ICC: 0.10.

Over the intervention period, students in the PAL intervention, had a favorably mean difference in change in aerobic fitness by 19.7 m (*p* < 0.001, intra class correlation (ICC) for school = 0.13) compared with students in the control group (Fig. 3A & B, αpath). In comparison, students in the DWBH intervention had a unfavorably mean difference in change and decreased their aerobic fitness by 11.5 m compared with their control school counterparts (*p* < 0.030, ICC for school = 0.13; Fig. 4A & B, αpath).

### 3.1 Mediation effects

Aerobic fitness satisfied all steps for mediation in the PAL intervention (Fig. 3A & B). For numeracy, aerobic fitness partially mediated the intervention effect by 28% from a total effect (c path) of 1.73 points (95% CI: 1.13 to 2.33) to a natural direct effect (c’ path) of 1.24 points (95% CI: 0.58 to 1.91; Fig. 3A). When examining the mediation effect on reading, aerobic fitness fully mediated the intervention effect, with the total effect (c path) of 0.89 points (95% CI: 0.15 to 1.62) reduced to the natural direct effect (c’ path) of 0.40 points (95% CI: −0.48 to 1.28) (Fig. 3B). The pattern of results from the main mediation analysis did not change when the analysis was rerun stratified by gender (data not shown).

Aerobic fitness did not mediate the effect of the intervention on academic performance in the DWBH intervention, as the natural direct effect (c’ path) was not reduced when compared with the total effect (c path; Fig. 4A & B).

## 4 Discussion

Whilst our results suggest that PA that improve aerobic fitness mediates the intervention effect on academic performance in the PAL intervention, no evidence supported this in the DWBH intervention. Specifically, the analysis reveals that aerobic fitness partially mediated the effect on numeracy performance, and fully mediated the intervention effect on reading performance, in the PAL intervention.

The results showing that aerobic fitness can mediate the relationship between PA and academic performance agree with some cross-sectional findings (Kyan et al., 2019, Lambourne et al., 2013, Visier-Alfonso et al., 2021) though there are conflicting results within the literature (Aadland et al., 2017, Kwak et al., 2009). The assumption that aerobic fitness may be a key factor in increasing academic performance among adolescents is supported by results from a longitudinal study where adolescents classified as aerobically fit had higher academic performance when compared with their aerobically unfit peers (Sardinha et al., 2016). Importantly, the same study also reported that students who were categorized as unfit at baseline, but improved their fitness during follow-up, observed a positive impact on academic performance (Sardinha et al., 2016).

Some mechanisms might explain why aerobic fitness mediated the effect of the intervention on academic performance in the PAL intervention. The increase in PA and aerobic fitness observed is associated with enhanced cerebral capillary growth, blood flow, and nerve cells in the hippocampus, which in turn are associated with learning and memory related to academic performance (Chaddock et al., 2011, Hillman and Biggan, 2017). Higher aerobic fitness can increase communications between neurons and integration of regions that support academic performance (Chaddock-Heyman et al., 2016). Research has also shown that PA and aerobic fitness enhance the synthesis of brain-derived neurotrophic factor, which is associated with increased volume of the hippocampus and improved memory (Cotman et al., 2007, Erickson et al., 2011). Hence, the indirect impact of the intervention on academic performance via aerobic fitness might be due to the positive relationship between aerobic fitness and the physiology of the brain.

We cannot disentangle the effect of the different components in the PAL intervention; the three components in tandem may explain the mediation role of aerobic fitness on the intervention effect on academic performance. The teachers were encouraged to perform activities of moderate to vigorousintensity, which in turn could have enhanced the student’s aerobic fitness. Further, if we assume that most students in Norwegian lower secondary schools learn reading and numeracy skills through screen-based devices and lessons in the traditionally sedentary form, it is plausible that the components in the PAL intervention, in addition to enhancing aerobic fitness, also resulted in students being more focused in these learning situations and therefore taking better advantage of the lessons (Daly-Smith et al., 2018, Norris et al., 2020, Sneck et al., 2019).

The context of the components in the PAL intervention may explain why the mediation effect differed between numeracy and reading performance. As the structure and material of numeracy lessons make it suitable to incorporate into the PAL components, it is plausible that the partial mediation effect on numeracy is because the students practised numeracy related task while being active, thus, increased numeracy performance could not solely be explained by increased aerobic fitness. In comparison, the reading curriculum is more difficult to incorporate into the PAL components, which makes the argument that the intervention effect on reading was a result of their increase in aerobic fitness and not only the change in PA.

In the DWBH intervention, aerobic fitness did not mediate the intervention effect on academic performance. It is reasonable to believe that the lack of mediation is connected to the lack of significant intervention effect on PA and aerobic fitness. Therefore, the positive intervention effects we found on academic performance among students in the DWBH intervention are likely explained by other factors. We can only speculate about these factors. However, it is plausible that the self-selected activities chosen in the DWBH intervention may minimize fatigue and boredom, and lead to higher levels of self-efficacy, which in turn could optimize the students’ academic performance (Fedewa and Ahn, 2011). Another possible explanation is that the varied PA provided throughout the curriculum can enhance enjoyment related to academic subjects and therefore stimulate higher motivation and engagement with theoretical subjects. The activities could also encourage students to cooperate with classmates, employ strategies, and adapt to changing task demands, which may create a more stimulating learning environment.

## 5 Strengths and limitations

The large sample size and cluster RCT study design are among strengths of this study as we can add causal evidence to the current literature. However, strong assumptions need to be met when conducting mediation analysis. To demonstrate causal pathways of the intervention effect, the intervention–outcome, intervention–mediator, and mediator–outcome must be unconfounded (VanderWeele, 2016). Even though ScIM was a cluster RCT and schools were randomly assigned to one of three groups, we cannot warrant that the intervention–outcome and intervention–mediator are unconfounded. Importantly, the students were not randomized to receive or not receive the mediator, thus the mediator–outcome may still be confounded. Potential confounders not measured in the study includes pubertal development and cognition. Additionally, we cannot exclude the possibility of random measurement error affecting our results. Even though our measurement of aerobic fitness is valid in this age group, students were meant to run to voluntary exhaustion, and whether the students worked hard enough to get a valid test was subjectively judged by trained test personnel. Peak oxygen consumption (VO2peak) would probably be more sensitive to changes in aerobic fitness than the shuttle run test, but those measurements require more resources (Aadland et al., 2018), which we did not have in our study. Random measurement error in the measurement of PA and aerobic fitness may lead to regression dilution bias, which biases the estimates of regression models coefficients towards the null. Furthermore, even though we used a standardized national test for measuring academic performance, the validity of the test is unknown, which could affect the standard error of the estimates and widen the corresponding confidence intervals. Despite the importance of highlighting how statistical assumptions and random measurement error could have led to an underestimation of the pathways between the mediator and the outcome, it does not solely invalidate our findings. Finally, in line with most school-based PA interventions, the ScIM study did not include a non-active control group. Control schools performed the mandatory amount of PE; hence our findings need to be interpreted as the effects of additional school-based PA, and not of the individual effects of PA per se.

## 6 Implications and future perspective

Although the potential mediator confounding and measurement errors entail that the results presented in this study need to be interpreted with caution, the findings suggest that in school-based interventions, physical activities of an intensity that increases aerobic fitness should be emphasized when we are aiming to increase adolescents’ academic performance. As school-based PA interventions in general often consist of several different components, it is in many cases unclear whether it is one specific component or the combination of several that is necessary for the effects observed. Future research should focus on specific components and intensities of PA, which may help reduce the intervention length and costs, and provide more nuanced knowledge of how school-based PA interventions affect academic performance.

## 7 Conclusion

With its cluster randomized design and corresponding results, our study adds causal evidence of the potential mechanism on how school-based intervention can affect academic performance. If aiming to increase academic performance, school-based PA interventions that leads to increased aerobic fitness may be particularly beneficial. Further investigation is needed to identify the pathways through which interventions focusing more on the social aspect of PA rather than dose and intensity, can influence academic performance.

## CRediT authorship contribution statement

**Runar Barstad Solberg:** Project administration, Conceptualization, Data curation, Formal analysis, Writing – original draft, Writing – review & editing. **Jostein Steene-Johannessen:** Conceptualization, Supervision, Writing – review & editing. **Morten Wang Fagerland:** Analysis, Writing – review & editing. **Sigmund A. Anderssen:** Writing – review & editing. **Sveinung Berntsen:** Writing – review & editing. **Geir K. Resaland:** Writing – review & editing. **Esther M.F. van Sluijs:** Conceptualization, Supervision, Writing – review & editing. **Ulf Ekelund:** Writing – review & editing. **Elin Kolle:** Conceptualization, Supervision, Writing – review & editing.

## Declaration of Competing Interest

The authors declare that they have no known competing financial interests or personal relationships that could have appeared to influence the work reported in this paper.
